# The effects of Bleomycin A5 on infantile maxillofacial haemangioma

**DOI:** 10.1186/1746-160X-7-11

**Published:** 2011-07-07

**Authors:** Quan-feng Luo, Fu-yun Zhao

**Affiliations:** 1 Peking University School and Hospital of Stomatology, Department of Oral and Maxillofacial Surgery, #22 Zhongguancun Nandajie, Haidian District, Beijing 100081, P.R. China

## Abstract

**Objective:**

To examine the effects of bleomycin A5 on infantile maxillofacial haemangiomas.

**Methods:**

Bleomycin A5 was given by multiple intralesinoal injections and the dosage was given according to the age of the patient and size of the lesion. Parts of patients were accompanied by prednisone treatment(2-5 mg/kg, po, QOD.

**Results:**

All the haemangiomas involuted completely after treated with bloemycin A5 with better recovery of skin color and less scar forming in small haemangiomas.

**Conclusion:**

Infantile haemangioma could be effectively treated with bleomycin A5 without serious side effects.

## Introduction

Infantile hemangiomas are the most common tumor of infancy, which are benign vascular proliferations composed of densely packed capillaries with endothelial cells and pericytes expanding in a lobular pattern. In contrast to vascular malformations, infantile hemangiomas are usually absent or inconspicuous at birth and are characterized by a remarkably rapid postnatal proliferation and slow spontaneous involution. Although infantile haemangiomas can involute spontaneously, it is still difficult to predict the progression of some infantile haemangioma, even small lesions may result in major aesthetic handicap at certain sites, especially for the big infantile haemangiomas (> 4 cm), as it may develope to the extant of maxillofacial deformity and follow with complications (usually bleeding, ulceration, or obstruction). Therefore, some clinicians suggest that interfering in infantile haemangioma should be at the early stage[1-3]. Conservative therapies for infantile haemangiomas include pharmacotherapy, laser therapy and consulting doctors regularly.

Bleomycin (BLM, also known as Blenoxane) was first isolated as a Cu^2+^-containing glycooligopeptide antibiotic from the culture medium of streptomyces verticullust. It was soon found to be an anticancer agent and has ever since become one of the most widely used anticancer drugs[4-6].

New application of bleomycin A5 was found recent years, that it was also used in treating haemangioma[1-3]. Conrad Pienaar and his colleagues treated hemangioma with a standard injection of bleomycin of 0.3 to 0.6 mg/kg per injection. 73% patients had a response rate greater than 75% reduction in size of the hemangioma. None of the patients in their study received corticosteroids. Only was bleomycin A5 injected in the local site, no other drugs were used. Other scholars achieved similar results that bleomycin A5 was effective in treating haemangioma.

We used bleomycin A5 as sclerosing agent for infantile haemangiomas for more than 15 years, prednisone treatment accompanied with bleomycin A5 according to the patient's age and the size of haemangioma. In the present study, we reviewed the effects of bleomycin A5 on infantile haemangiomas treated during 1997-2005 in Peking University Hospital of Stomatology.

## Materials and methods

### Patients

**Figure 1:**
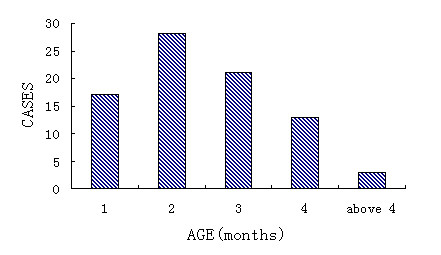
**Age of first consultation**.

**Figure 2:**
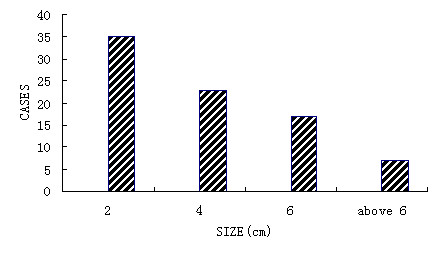
**Distribution of hemangioma size**.

A total of 82 cases of infantile haemangioma treated with bleomycin A5 during 1997-2005 in Peking University Hospital of Stomatology was reviewed. The patients (male 34 cases and female 48 cases) were presented within their first year of life with the majority before 4 months old (Figure 1). The size of the haemangioma is mostly less than 6 cm (Figure 2).

### Diagnosis of infantile haemangioma

Infantile haemangioma was diagnosed by clinical evaluation of age, and appearance and development of the lesion, combined with ultrasonography or magnetic resonance imaging (MRI) or color Doppler. Attention was paid to differentiate haemagioma from vascular malformation. All of the haemangiomas were located at head, face or neck.

### Application of bleomycin A5

The sclerosing mixture is composed of 5 ml 2% lidocaine, 5 mg dexameson and 8 mg bleomycin A5. The mixture was multiplely injected with 5-gauge needle through the places close to the lesion for avoiding bleeding until the hemangioma became pale. The bleomycin was injected in a radial fashion. The dosage was given according to the age of the patient and size of the lesion. Generally, lesion of 2 cm diameter was given 1 mg bleomycin A5 per time and total 5 times would be enough. For the patient with bigger haemangioma, the amount of bleomycin A5 was usually less than 3 mg per time. The amount of bleomycin A5 is usually less than 0.5 mg when the baby is less than 3 month, the amount less than 1.5 mg before 6 months, less than 2 mg before 1 year old, less than 2.5 mg before 2 years old [Table 1].

**Table 1:** The dosage according to lesion size and patient's age

	Lesion size(diameter, cm)				Age(month)			
	0-2	2-4	4-6	> 6	< 3	< 6	< 12	< 24

***Amount*** ***(mg)***	1	2	3	> 3	0.5	1.5	2	2.5

The interval of injection was 3 to 4 weeks with total times less than 7 times during one therapeutic period. Another treatment periods started 3 months later if further treatment was necessary. The total quantity of bleomycin A5 for a child should be less than 40 mg in one treatment periods.

Prednisone treatment was used according to the patient's age and the size of the lesion. If the age was less than 7 months, prednisone was given by mouth(2-5 mg/kg/day, QOD, for one month). If the lesion was more than 4 cm, prednisone treatment lasted for 2 periods. Prednisone was usually given at the third month and the sixth month after birth.

### Evaluation of the effects of bleomycin A5

The size and blood flow of the hemangioma were evaluated with color ultrasonography every two months. The change of the lesion skin color and scar forming was also evaluated every two months.

Satisfaction of family members was determined according to the final appearance of patient.

## Results

The effects of bleomycin A5 on infantile haemangiomas were divided into three degrees and sumarised in table 2. Degree I: the lesion involuted completely with normal function. The color of the lesion skin and muccal were also recovered to normal. Degree II: the lesion involuted completely but scar formed or the color was not recovered to normal. Degree III: the lesion was only partially involuted.

**Table 2:** Lesion's involution and family's satisfaction

Size(cm)	0-2			2-4			4-6			Above 6		
Degrees	I	II	III	I	II	III	I	II	III	I	II	III

Cases	21	14	0	8	15	0	5	12	0	0	7	0

Percentage(%)	60	40		34.8	65.2		28.8	71.2			100	

*Satisfaction(%)*	88.6			82.6			88.2			57.1		

As sumarised in Table 2, all the haemangiomas involuted completely after treated with bloemycin A5. However, the haemangiomas less than 2 cm were easier recovered to normal skin color with less scar forming, comparing with the haemangiomas more than 2 cm.

Size/colour and blood flow changes. The lesion usually begins to decrease after the third time, the height of lesion first decreases with colour fading, then the diameter decreases after 4-5 times injection. Blood flow declines after 4-5 times injection too, but blood flow signal disappears earlier than colour (usually after 5-6 times injection whose diameter is less than 4 cm).

**Figure 3:**
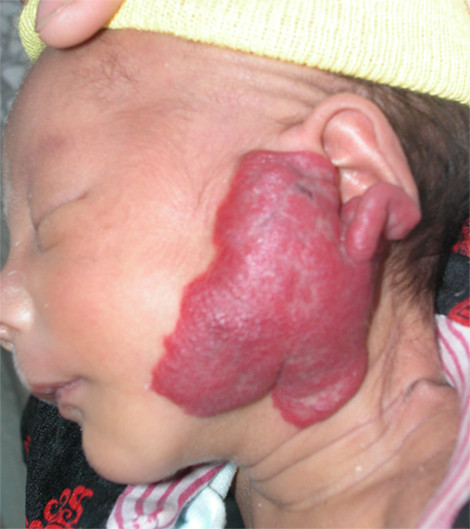
**This boy presented at 40 days of age with a hemangioma in the left parotid area**. Bleomycin A5 was given once every 4 weeks for a total of 7 treatments.

**Figure 4:**
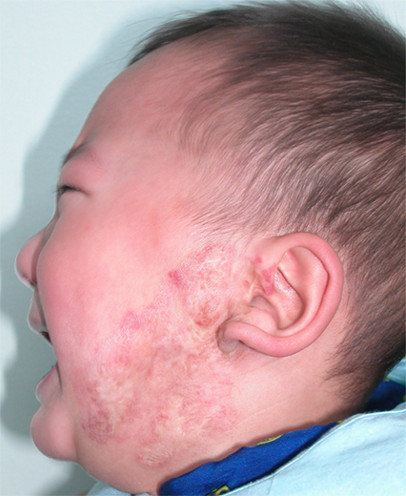
**The hemangioma had completely involuted one year later**. The overall response was Scale II.

**Figure 5:**
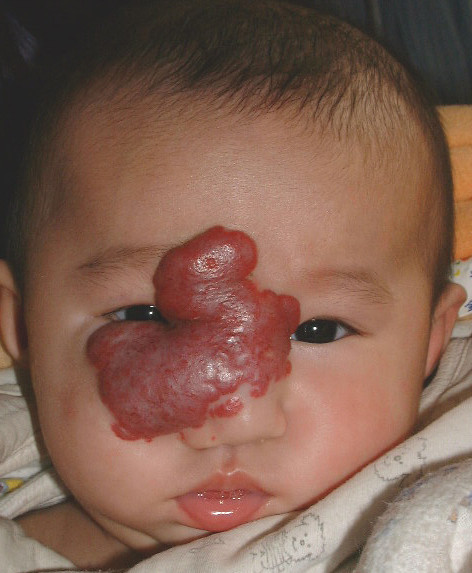
**This girl had a hemangioma at the center of her face that involved the nose, right eyelid, and bilateral medial canthi**.

**Figure 6:**
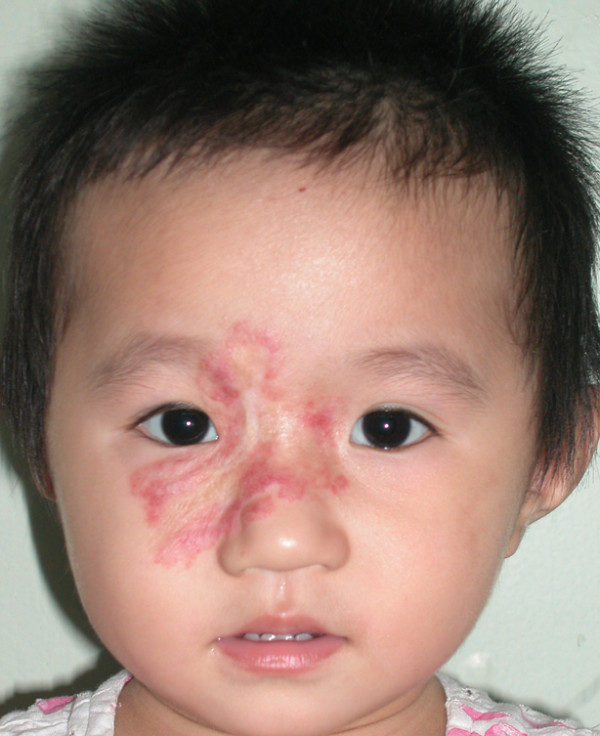
**The hemangioma involuted one year and five months after bleomycin treatment**. The overall response was Scale II.

**Figure 7:**
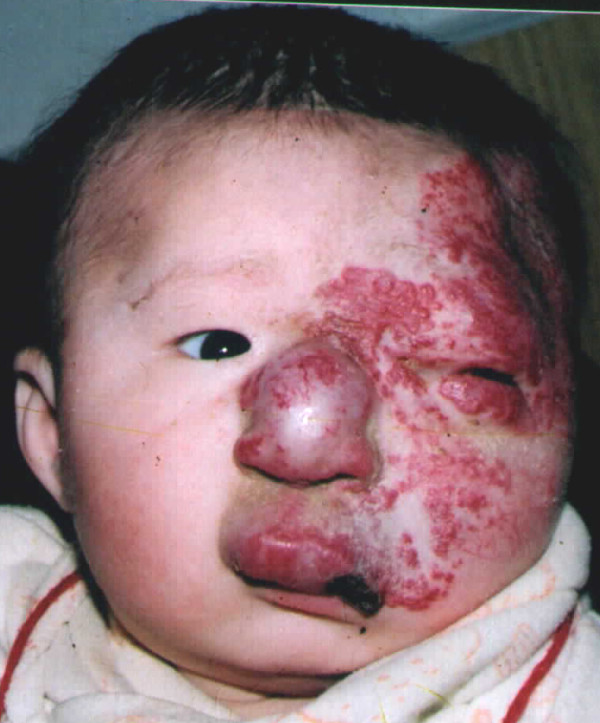
**A large hemangioma was present on the left face, involving the eyelid, nose, and upper lip**. Bleomycin A5 was given once a month for 7 months. Four months later, treatment was resumed again. The total treatment period was 2 years and 4 months.

**Figure 8:**
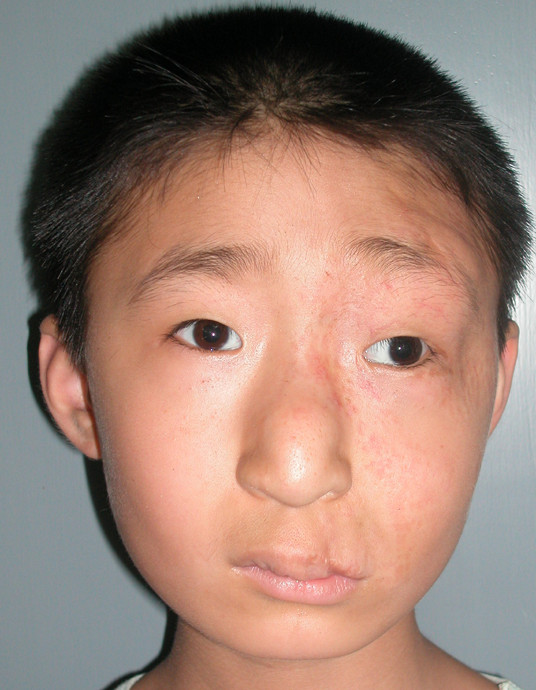
**Three years later, the hemangioma had completely involuted, with few scars, the overall was Scale I**.

The percentage of satisfaction was also higher in the patients with haemangioma less than 6 cm, but percentage of of dissatisfaction was higher with haemangioma more than 6 cm [Table 2]. Typical cases were given in Figures 3, 4, 5, 6, 7 and 8.

Complications included edema, ulceration, gastrointenstinal side effects, and others [Table 3]. Edema emerged about 4 hours after the injection and reached maximal degree about two days later. The edema generally disappeared about 15 days later. Ulceration happened in the surface of the haemangiomas and healed mostly in 15 days with scar forming.

**Table 3:** Complications during the usage of Bleomycin A5

Complication	Edema	Gastrointenstinal	Ulceration	Pneumonic fibrosis	Others
***Cases***	82	5	6	0	0

Gastrointenstinal side effects included nausea and lack of appetite and often happened one day later, disappeared three days later.

## Discussion

Infantile haemangiomas are usually small lesions and can involute spontaneously. However, some infantile haemangiomas will not involute and even develope to destroy the organ founction with deformity. Clinically there is no good method to predict the process of infantile haemangioma. It is still controversial whether the infantile haemangioma should be treated or not. According to our clinical experience, we suggested that infantile haemangioma should be treated at early stage to prevent the haemangioma from proliferation.

There are several well-established treatments for infantile haemangioma. The major options include corticosteroids (either intralesional or systemic corticosteriods), interferon-α, laser therapy, cryotherapy, and surgical excision [7-10]. We used Bleomycin A5 as sclerosesing agent to modulate angiogenesis of the infantile haemangiomas, and achieved good effects.

Sometime we used bleomycin A5 accompanying by prednisone. We observed that infantile haemangioma usually proliferated rapidly before the postnatal seventh month, especially in the third and sixth month. Although bleomycin A5 can inhibit the haemangioma proliferation effectively, but the dosage was restricted, therefore the drug quantity given in one time may be deficient for big haemangioma(> 4 cm). Another clinical phenomenon which should be paid attention to was that the rapid proliferation in a few of infantile haemangioma can't be controlled at once after bleomycin injection. All above were the reasons that we sometime used prednisone as adjuvant therapy. This is different from other scholars [1-3]. All infantile haemangioma were cured through this way in our hospital.

The pathogenesis of haemangioma is due to the proliferation of vascular endothelial cells. Bleomycin acts on S stage of cell cycle to snip DNA chain during cell mitosis and disturb the cell proliferation. Therefore, the effects of Bleomycin A5 on infantile haemangioma is believed to destroy the proliferation of vascular endothelial cells. The onset of involution is usually heralded by a change in color from bright red to purple or gray after treated with Bleomycin A5 for several times. Small haemangiomas (< 2 cm) would be effectively treated within 5 times of injection with total quantity less than 10 mg of Bleomycin A5. However, big haemangioma (> 4 cm) usually needs more than 8 times of injection with total quantity more than 16 mg of Bleomycin A5. The therapeutic effect was related to the lesion size and the dosage of bleomycin A5 being given.

Some complications occurred during the treatment. Edema was the most common complication, followed by ulceration. However, serious side effects would rarely occur due to the lower dosage in this treatement.

Conclusively, we reviewed the effects of bleomycin A5 on 82 cases of infantile haemangiomas and found that all the haemangiomas involuted completely after treated with bloemycin A5 with better recovery of skin color and less scar forming in small haemangiomas. The results suggested that infantile haemangioma could be effectively treated with bleomycin A5 without serious side effects.

## Consent

Written informed consent was obtained from the patient for publication of this case report and accompanying images. A copy of the written consent is available for review by the Editor-in-Chief of this journal.

## Competing interests

The authors declare that they have no competing interests.

## Authors' contributions

QFL conceptualized the paper. QFL and FYZ drafted and edited the manuscript, the treatment were performed by them too. All authors have read and approved the final manuscript.
